# Study Features and Response Compliance in Ecological Momentary Assessment Research in Borderline Personality Disorder: Systematic Review and Meta-analysis

**DOI:** 10.2196/44853

**Published:** 2023-03-15

**Authors:** Antonella Davanzo, Delfine d´Huart, Süheyla Seker, Markus Moessner, Ronan Zimmermann, Klaus Schmeck, Alex Behn

**Affiliations:** 1 Escuela de Psicología Pontificia Universidad Católica de Chile Instituto Milenio para la Investigación en Depresión y Personalidad Santiago Chile; 2 Department of Child and Adolescent Psychiatry Research University Psychiatric Clinics University of Basel Basel Switzerland; 3 University Hospital Heidelberg Center for Psychotherapy Research Heidelberg Germany

**Keywords:** borderline personality disorder, ecological momentary assessment, compliance, study design features, e–mental health, mobile phone

## Abstract

**Background:**

Borderline personality disorder (BPD) is characterized by frequent and intense moment-to-moment changes in affect, behavior, identity, and interpersonal relationships, which typically result in significant and negative deterioration of the person’s overall functioning and well-being. Measuring and characterizing the rapidly changing patterns of instability in BPD dysfunction as they occur in a person’s daily life can be challenging. Ecological momentary assessment (EMA) is a method that can capture highly dynamic processes in psychopathology research and, thus, is well suited to study intense variability patterns across areas of dysfunction in BPD. EMA studies are characterized by frequent repeated assessments that are delivered to participants in real-life, real-time settings using handheld devices capable of registering responses to short self-report questions in daily life. Compliance in EMA research is defined as the proportion of prompts answered by the participant, considering all planned prompts sent. Low compliance with prompt schedules can compromise the relative advantages of using this method. Despite the growing EMA literature on BPD in recent years, findings regarding study design features that affect compliance with EMA protocols have not been compiled, aggregated, and estimated.

**Objective:**

This systematic meta-analytic review aimed to investigate the relationship between study design features and participant compliance in EMA research of BPD.

**Methods:**

A systematic review was conducted on November 12, 2021, following the PRISMA (Preferred Reporting Items for Systematic Reviews and Meta-Analyses) and MOOSE (Meta-analyses of Observational Studies in Epidemiology) guidelines to search for articles featuring EMA studies of BPD that reported compliance rates and included sufficient data to extract relevant design features. For studies with complete data, random-effect models were used to estimate the overall compliance rate and explore its association with design features.

**Results:**

In total, 28 peer-reviewed EMA studies comprising 2052 participants were included in the study. Design features (sampling strategy, average prompting frequency, number of items, response window, sampling device, financial incentive, and dropout rate) showed a large variability across studies, and many studies did not report design features. The meta-analytic synthesis was restricted to 64% (18/28) of articles and revealed a pooled compliance rate of 79% across studies. We did not find any significant relationship between design features and compliance rates.

**Conclusions:**

Our results show wide variability in the design and reporting of EMA studies assessing BPD. Compliance rates appear to be stable across varying setups, and it is likely that standard design features are not directly responsible for improving or diminishing compliance. We discuss possible nonspecific factors of study design that may have an impact on compliance. Given the promise of EMA research in BPD, we also discuss the importance of unifying standards for EMA reporting so that data stemming from this rich literature can be aggregated and interpreted jointly.

## Introduction

### Background

Borderline personality disorder (BPD) is a serious mental disorder affecting approximately 1% of the adult population, 12% of the adult outpatient population, and 22% of the adult inpatient population [1]. BPD is characterized by frequent and intense moment-to-moment changes in affect, behavior, identity, and interpersonal relationships, which typically result in a significant deterioration of the person’s overall functioning and well-being [2]. It is 1 of the 10 personality disorders listed in the latest edition of the Diagnostic and Statistical Manual for Mental Disorders (DSM)–Fifth Edition [3]. Even though new international diagnostic classification systems, such as the International Classification of Diseases 11th Revision, removed specific types of personality disorder in favor of a general diagnosis of personality disorder, BPD was retained as a specific pattern [4].

According to established diagnostic criteria, BPD is marked by severe, unpredictable, and intense feelings; vulnerability to perceived rejection or alienation in the context of interpersonal hypersensitivity; behavioral dysregulation involving suicidality and often impulsiveness; and an impaired sense of self, usually contributing to identity disturbance, a poor sense of direction, poor self-esteem, and cognitive disorders [5]. All these areas of core BPD psychopathology are typically characterized by high instability over time [6,7]. Because of the high variability in BPD symptoms, it is desirable to study moment-to-moment variability to capture the true extent of the disorder. The ecological momentary assessment (EMA; sometimes referred to as experience sampling) [8] uses systematic and often very frequent self-report diaries for evaluating context, symptoms, stressors, and other factors as they occur in everyday life and in nonlaboratory or research settings.

### EMA Studies

EMA studies are characterized by the use of repeated, often very frequent assessments that are delivered to participants in real-life settings and in real time using handheld devices capable of registering responses to short self-report questions in daily life. These repeated real-time measurements provide many methodological advantages over conventional assessment strategies, as highlighted in the study by Shiffman et al [9]. First and foremost, in the setting of highly and rapidly variable phenomena, recall biases encountered in conventional retrospective survey methods are diminished by the momentary evaluation of participants’ current experiences or behaviors. Second, measurements taken *in the moment* and *in the context* in natural settings yield information that is more pertinent to the existing social or physical contexts, providing information that is more ecologically valid. Third, the daily intensive repeated measurements capture within-day, within-person behavior as well as experience alterations over time, enabling studies of immediate causes and effects of behavior in real time [10].

### EMA for the Study of BPD Symptoms

The EMA methodology is being increasingly used to study psychopathology processes in BPD, mainly because these are considered very unstable and variable over time, thus benefiting from frequent sampling schedules. Given the variability of BPD symptoms, several momentary ratings that minimize retrospective bias and have strong ecological validity can be useful in modeling the dynamic core psychopathology features of the disorder [11]. This has led to important scientific and clinical discoveries by expanding our understanding of diagnosis, symptomatic variability, and disease mechanisms [12]. For example, the studies by Gordon and Laws [13] and Sadikaj et al [7] reported using EMA that patients with BPD exhibit high emotional variability in their responses to interpersonal stressors. The study by Stiglmayr et al [14] found that dissociation was correlated with distress in patients with BPD, which varied over time. The study by Zeigler-Hill and Abraham [15] reported interesting findings revealing that patients with BPD show increased self-esteem variability, especially in response to stressful interpersonal situations with peers. The study by Links et al [16] examined the link between emotional disturbances (specifically, the intensity of negative mood, mood amplitude, mood dyscontrol, and mood triggering) and suicide attempts. Therefore, EMA appears to be an appropriate tool for measuring the variability in BPD symptoms. Another vital contribution of EMA to BPD research is the possibility of studying the causes and effects of behavior in real time [17]. This could be useful for the therapeutic management of risk situations [18], as has been developed in the dialectical behavior therapy chain analysis technique [19].

Even though BPD is characterized by severely debilitating symptoms, participant compliance with EMA protocols appears to be relatively high, suggesting that symptom severity may not per se affect compliance [20]. It is possible that in the case of BPD, the impact of severe symptoms on compliance is counteracted by a beneficial effect of testing reactivity. Testing reactivity refers to the extent to which the behavior of interest is modified by momentary questions [21]. Few studies have examined the specific effects of testing reactivity on BPD symptom severity and intensity, but available models suggest that through the processes of active reflection and self-monitoring, social desirability, or feedback processes, the intensity of symptoms may decrease as a result of unusual attention on target symptoms [22]. In fact, active and frequent self-monitoring may be a generic active ingredient across a variety of evidence-based psychosocial interventions for BPD (eg, the use of diary cards to identify triggers and patterns of affective dysregulation in dialectical behavior therapy) [23].

### The Importance of Ensuring Compliance in EMA Studies

EMA studies offer numerous advantages over traditional retrospective reporting in the study of highly variable phenomena over time; however, they typically place a high demand on participants, which can, in turn, lead to low response compliance. Low response compliance occurs when the ratio of answered prompts in relation to the theoretical maximum number of prompts planned in the study is low and has a direct bearing on data quality [16]. If compliance is low, sparsely collected data are unlikely to be a valid measure of the intensity and variability of the phenomena of interest measured in daily life, eliminating all relative advantages of using experience sampling. Even though missingness is a frequent occurrence in EMA studies, when compliance falls critically low or when patterns of noncompliance occur systematically at specific measurement occasions (eg, a participant always misses prompts delivered when she is experiencing intense emotional distress), the validity of the study is compromised [14]. Valid inferences about moment-to-moment experiences reported by participants using EMA methodologies must yield sufficient data to be robust and valid. Therefore, EMA studies typically include different design features and use strategies for enhancing compliance with the study protocol. For instance, it is common for EMA researchers to offer some kind of financial compensation to participants, which may even be contingent on a certain percentage of compliance (eg, the study by Berenson et al [24]). Other strategies include briefing the participants about the intensive nature of the study and even arranging telephone calls when a participant engages in increased noncompliance [25].

### Design Features of EMA Studies Affecting Compliance

Two recent meta-analyses examined design features that broadly affect response compliance in EMA research. The study by Wrzus and Neubauer [26] examined this issue in EMA research across research fields by sampling 477 articles including 677,536 participants. They found that most EMA studies involved 6 assessments per day and lasted 7 days, although the number of assessments was not related to response compliance. Across studies, a compliance rate of 79% was obtained for participants with BPD, and providing financial incentives significantly increased response compliance. Other design features had little or no effect on compliance. The authors also emphasized the high heterogeneity in study design and reporting and called for more standardized procedures for EMA planning and reporting. The study by Vachon et al [27] explored the issue of compliance with EMA protocols in studies that included different mental disorders. The authors sampled data from 8013 participants. Typically, EMA designs included an average of 6.9 assessments per day for an average of 11.2 days. The study by Vachon et al [27] found that compliance rates were significantly lower for studies that included a higher proportion of male participants and participants with psychotic disorders. Compliance seemed to increase when researchers used fixed sampling schemes (ie, prompts were delivered in preplanned intervals across the day), used higher financial incentives, and spaced successive assessment occasions more over time (ie, less intensive protocols). The study by Smyth et al [28] covered a different aspect and investigated the relationship between the intention to participate in EMA studies and aspects of design features (such as prompt frequency and study duration). They found that willingness to participate was higher in simpler and less intense protocols and suggested that future EMA studies explore how different combinations of design features may influence participation. Taken together, previous studies underline the vast heterogeneity in EMA planning and reporting and call for unified standards.

### Aim of This Study

EMA appears to be particularly well suited for the study of basic BPD psychopathology. To capture all the benefits of using this methodology to advance the field and construct more robust and nuanced dynamic psychopathology models, data must be of the highest quality, and compliance with study protocols is a crucial component of data quality in EMA research. However, to date, there are no systematic reviews and meta-analyses examining compliance in EMA research of BPD psychopathology, including the role of standard design features in enhancing compliance. In this setting, this review had three specific goals: (1) to provide a narrative overview of the EMA methodologies used to investigate dynamic BPD psychopathology; (2) to obtain and report overall compliance rates and quantitatively examine the effect of regular study design features (ie, the number of daily prompts, measurement period of the study, number of items in each prompt, etc) and study procedures (ie, the use of financial incentives, the device type, etc) on compliance rates; and (3) to optimize the advantages of EMA techniques in investigating BPD, for which this study provides recommendations for future studies that will use mobile devices to collect real-time self-reported data from BPD participants.

## Methods

This systematic review and meta-analysis were conducted in accordance with the PRISMA (Preferred Reporting Items for Systematic Reviews and Meta-Analyses; Multimedia Appendix 1) standards [29] as well as the MOOSE (Meta-Analyses of Observational Studies in Epidemiology) guidelines [30].

### Literature Search

Bibliographic literature searches were conducted in 4 electronic databases (PsycINFO, PubMed, Scopus, and Web of Science) on November 12, 2021, without any time or language restrictions. Keywords based on Medical Subject Headings terms were used to identify peer-reviewed journal articles reporting on the use of EMA among the population with BPD. The search terms used in the individual databases are presented in Table S1 in Multimedia Appendix 2.

The search results for both the systematic review and meta-analysis were screened independently by 3 authors (AD, DH, and SS) using Covidence (Veritas Health Innovation) [31], a web-based review management tool for screening and data extraction. The screening process was carried out in 2 stages: title and abstract stage and full-text stage. Discrepancies were resolved through discussion according to the eligibility criteria previously defined.

### Inclusion and Exclusion Criteria

#### Inclusion and Exclusion Criteria for the Systematic Review

Studies had to meet the inclusion criteria shown in Textbox 1 to be included in the systematic review.

Studies that involved participants without BPD or healthy participants in addition to participants with BPD were included only if separate analytic or descriptive results were presented for the BPD or BPD feature subgroup.

Studies that met the exclusion criteria shown in Textbox 2 were not included.

**Inclusion criteria for the systematic review.**

- Should be an empirical study (not a review or comment)
- Should use ecological momentary assessment strategies or experience sampling methods, including diary methods
- Should use mobile or handheld technologies for ecological momentary assessment or experience sampling method data collection (eg, cell phones, PDAs, and smartphones)
- Should include participants diagnosed with borderline personality disorder (BPD) or BPD features (ie, ≥1 of the 4 core areas of BPD: interpersonal problems, affect dysregulation, cognitive self-dysregulation, and behavioral dysregulation)
- Should assess BPD or BPD features according to the *Diagnostic and Statistical Manual for Mental Disorders* or the International Classification of Diseases

**Exclusion criteria for the systematic review.**

- Studies did not use any electronic, wearable, or mobile technology
- Studies used call-based (ie, phone interview) methods instead of prompts for data collection
- Studies did not collect data in ecological or free-living natural setting (eg, studies collected data in artificial settings such as laboratories)

#### Inclusion and Exclusion Criteria for the Meta-analysis

Studies included in the systematic review were entered into the meta-analytical part of this study if they fulfilled the following inclusion criterion: sufficient information was available that allowed the estimation of an average compliance rate (ie, the proportion of answered prompts) for the BPD or BPD feature subgroup.

Thus, studies that did not report compliance rate or in which compliance rate could not be extracted for the BPD or BPD feature subgroup were excluded from the meta-analytical part of the study.

### Data Extraction

A total of 3 authors (AD, DH, and SS) independently extracted the study characteristics as well as EMA design features and procedures from each included study. The extracted study characteristics were location, BPD sample size, mean age of the BPD sample (in years), sex (percentage of female participants), and dropout rates of participants with BPD (in percentage). Furthermore, the extracted EMA design features and procedures were study length (in days), daily prompting frequency, extra assessment (ie, if there was a daily extra measurement in addition to the prompts), number of items (ie, items to be answered in each prompt), average time to answer a prompt (in minutes), assessment window in minutes (ie, how long each prompt is available to be answered before it is considered missed), device type (ie, smartphone or others), sampling scheme (ie, time-based or self-initiated prompts), prompt scheme (ie, fixed or random prompts), and the incentive given (ie, fixed or incremental incentive). Finally, if possible, data on the compliance rate were extracted. If the compliance rate was not explicitly reported, the number of total prompts sent and the number of answered prompts were extracted from each study, for the BPD sample and (if available) the non-BPD sample. Discrepancies in author coding assignments were discussed and resolved until a consensus was reached. If needed, attempts were made to contact the authors of the studies with incomplete or insufficient data.

We were unable to obtain full data from 7 studies that did not report separate results for control and BPD groups [32-38], which were, therefore, excluded from our study.

### Quality Assessment

The methodologic quality of the included studies was independently rated by 3 authors (AD, DH, and SS) using the Newcastle-Ottawa Scale [39].

This tool was specifically developed for the quality assessment of nonrandomized studies in systematic reviews and meta-analyses. Using this tool, each study is judged on 8 items, which are categorized into three broad domains: (1) the selection of the study groups (comprising the items “Representativeness of the Exposed Cohort,” “Selection of non-Exposed Group,” “Ascertainment of Exposure,” and “Demonstration of Absence of Outcome at Start of Study”), (2) the comparability of the cohort groups (comprising the item “Comparability Based on the Design or Analysis”), and (3) the ascertainment of either the exposure or outcome of interest for case-control or control studies, respectively (comprising the items “Assessment of Outcome,” “Follow-up Long Enough for Outcomes to Occur,” and “Adequacy of Follow-up of Cohorts”). To rate the comparability of the study cohort groups, the Newcastle-Ottawa Scale requires the authors to predetermine 1 or 2 key control variables. We included age as the first control variable and sex as the second control variable. If 2 of the predefined control variables were met, the criterion “Comparability Based on the Design or Analysis’ was rated with 2 scores. For the items “Follow-up Long Enough for Outcomes to Occur” and “Adequacy of Follow-up of Cohorts,” the follow-up interval was defined as the time between the first EMA measurement point (ie, baseline) and the last EMA measurement point (ie, follow-up).

Each study was rated in terms of 8 criteria (7 criteria were scored 0 or 1, and 1 criterion was scored 0, 1, or 2), resulting in a maximum score of 9 for each study. Discrepancies in author coding assignments were discussed and resolved until consensus was reached. The quality of a study was assessed as *high* if 7 to all the 9 criteria were met, *moderate* if 4 to 6 criteria were met, and *low* if ≤3 criteria were met.

### Statistical Analyses

The “meta” package in R (version 4.0.2, R Foundation for Statistical Computing) was used for all analyses and plots [40]. We used a 2-sided significance level of *P*<.05 to indicate statistical significance.

First, compliance rates were calculated by dividing the total number of prompts delivered by the study by the number of answered prompts at the end of the study for the BPD sample in each study. We chose a random-effects model for assessing the effect sizes rather than fixed-effects models because significant variability between studies was assumed [41]. We assessed the between-study heterogeneity of the results by calculating the *Q* statistic, *I*^2^, and τ^2^ [42-44]. In general, *I*^2^ can be interpreted as follows: a proportion of 25% is assumed to indicate a low heterogeneity; 50%, a moderate heterogeneity; and 75%, a high heterogeneity [43].

A total of 4 categorical design features and procedures of EMA studies (ie, instrument, device type, quality rating, and extra assessment) were tested on the effects of compliance rates in the BPD sample using univariate analyses with random-effects models. Analyses testing the effects of continuous design features and procedures on compliance rate (ie, study length, prompts per day, and total quality score) were omitted, as the included subgroups contained <10 studies. As is true for primary studies, which require an appropriately large ratio of participants to form meaningful subgroups, meta-analyses require an appropriately large number of studies. Therefore, the use of meta-regression is generally not recommended when the number of studies is small (ie, n<10) [43].

## Results

### Study Selection

**Figure 1:**
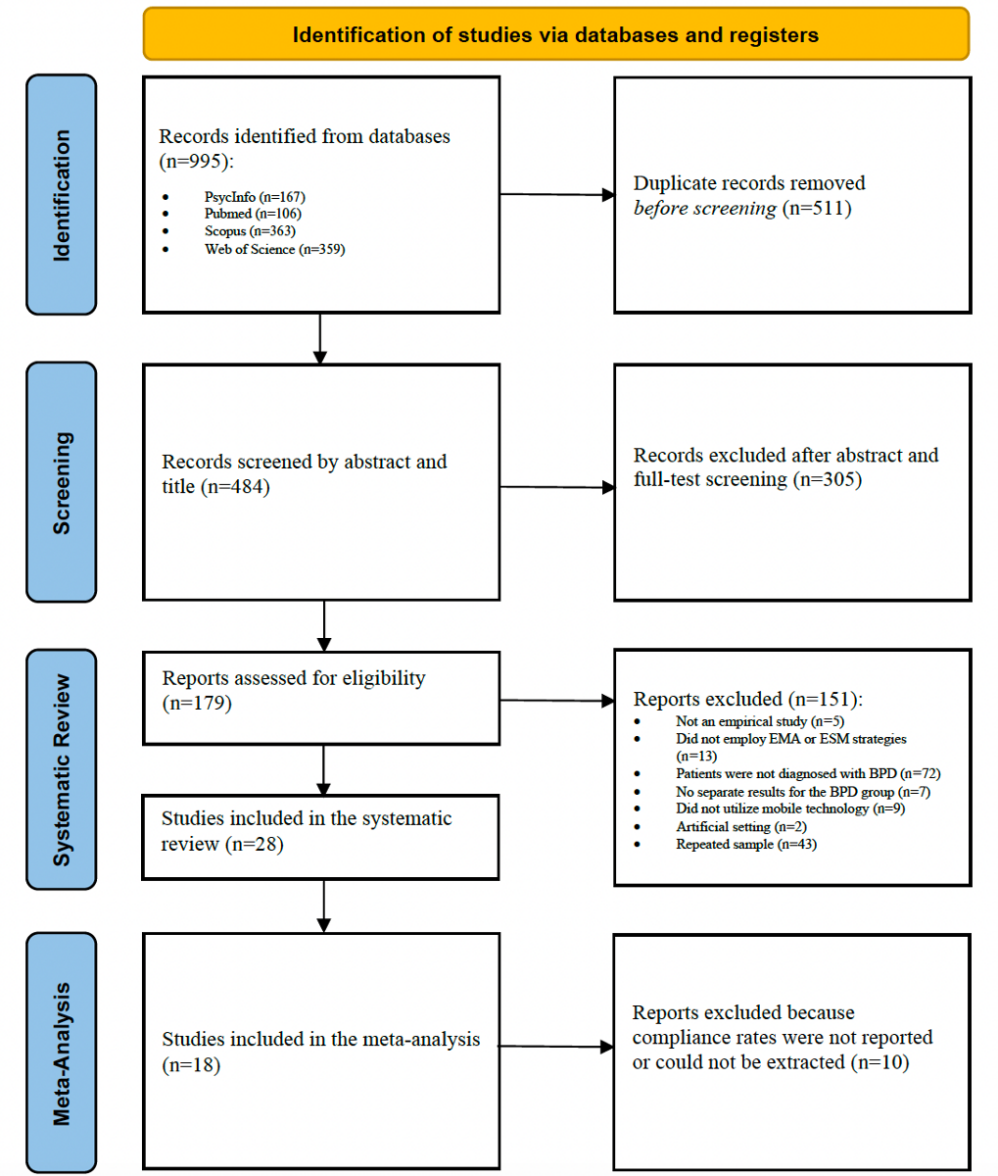
Flowchart of study inclusion. BPD: borderline personality disorder; EMA: ecological momentary assessment; ESM: experience sampling method.

In total, the systematic literature search revealed 995 potentially relevant articles. The screening and full-text assessment resulted in 28 peer-reviewed EMA studies [16,17,24,45-69] including a total sample of 2052 participants (N=1158 for the BPD group, 56.43%; N=894 for the control group, 43.57%; refer to Figure 1 for a flowchart of study inclusion). All studies examined BPD symptoms and selected participants based on either DSM criteria (26/28, 93%) or International Classification of Diseases criteria (2/28, 7%). For a complete list of the included studies and their characteristics, refer to Tables 1 and 2.

**Table 1:** Characteristics of the included studies (n=28).

Study	Publication year	Location	Sample size	Age (years), mean (SD)	Sex (female; %)	Dropout, (%)	Diagnostic criteria^a^	Clinical status
Andrewes et al [45]	2017	Oceania	107 BPD^b^	18.1 (2.7)	83.2	1.9	2 in DSM^c^	Outpatient
Berenson et al [24]	2011	United States	45 BPD and 40 CG^d^	33.5 (10.2)	76.5	8.9	5 in DSM	Outpatient
Briones-Buixassa et al [46]	2021	Europe	22 BPD and 42 CG	23.6 (5.1)	90	N/R^e^	2 in DSM	Outpatient
Chaudhury et al [47]	2017	United States	50 BPD	30.6 (11.0)	86	N/R	5 in DSM	Outpatient
Coifman et al [48]	2012	United States	65 BPD and 61 CG	32.3 (11.6)	82	0	5 in DSM	N/R
Ebner-Priemer et al [49]	2006	United States and Europe	50 BPD and 50 CG	31.3 (8.1)	100	N/R	1 in DSM	Outpatient
Ellison et al [50]	2020	United States	27 BPD and 15 CG	32.5 (11.3)	90.5	30	5 in ICD^f^	Outpatient
Hawkins et al [51]	2014	United States	N/R	41.0	68	N/R	5 in DSM	Outpatient
Houben et al [52]	2016	Europe	30 BPD and 28 CG	29.0 (1.6)	87	N/R	1 in DSM	Inpatient
Kaurin et al [53]	2020	United States	43 BPD and 164 CG	30.5 (6.8)	54	N/R	5 in DSM	Outpatient
Köhling et al [54]	2016	Europe	20 BPD and 21 CG	26.2 (6.5)	100	10	1 in DSM	Inpatient
Lane et al [55]	2016	United States	56 BPD and 60 CG	26.4 (7.1)	78.5	N/R	1 in DSM	Outpatient
Links et al [16]	2007	South America	82 BPD	33.5 (10.3)	82.9	N/R	5 in DSM	Outpatient
Moukhtarian et al [56]	2020	Europe	41 BPD and 57 CG	35.4 (11.4)	100	10	4 in DSM	Outpatient
Rizk et al [57]	2019	United States	38 BPD	28.6 (9.5)	100	N/R	5 in DSM	Outpatient
Santangelo et al [12]	2017	Europe	60 BPD and 60 CG	27.2 (7.0)	100	0	5 in DSM	Outpatient
Scala et al [58]	2018	United States	36 BPD and 18 CG	34.2 (12.4)	92	N/R	5 in ICD	Outpatient
Selby et al [59]	2021	United States	1974 BPD and 2726 CG	19.1 (1.8)	68.1	N/R	1 in DSM	Outpatient
Solhan et al [60]	2009	United States	58 BPD and 42 CG	32.3	93.1	N/R	1 in DSM	Outpatient
Southward et al [61]	2020	United States	8 BPD	21.6	3.05	N/R	5 in DSM	Outpatient
Stiglmayr et al [14]	2008	Europe	51 BPD and 91 CG	27.1 (6.7)	100	N/R	5 in DSM	Inpatient
Trull et al [17]	2008	United States	34 BPD and 26 CG	33.3 (12.4)	97.1	N/R	1 in DSM	Outpatient
Veilleux et al [62]	2021	United States	49 BPD and 50 CG	19.3 (2.1)	72.3	N/R	5 in DSM	Outpatient
Weise et al [63]	2020	Europe	43 BPD	15.5 (1.2)	95.4	N/R	1 in DSM	Outpatient
Wright et al [70]	2016	United States	5 BPD	N/R	N/R	N/R	5 in DSM	Outpatient
Wycoff et al [64]	2020	United States	54 BPD	26.2 (7.2)	81.5	N/R	2 in DSM	Outpatient
Zaehringer et al [65]	2019	Europe	26 BPD	33.4 (11.1)	100	15.4	1 in DSM	Outpatient
Zaki et al [66]	2013	United States	38 BPD and 42 CG	29.9 (10.6)	84	N/R	5 in DSM	Outpatient

^a^1=affect dysregulation, 2=behavioral dysregulation, 3=interpersonal instability, and 4=cognitive or self-disturbance*.*

^b^BPD: borderline personality disorder.

^c^DSM: Diagnostic and Statistical Manual for Mental Disorders.

^d^CG: control group.

^e^N/R: not reported.

^f^ICD: International Classification of Diseases.

**Table 2:** Ecological momentary assessment.

Study	Sampling scheme^a^	Study length (days)^b^	Extra assessment^c^	Prompts per day (n)^d^	Items (n)^e^	Required answer time (minutes)^f^	Assessment window (minutes)^g^	Device type	Incentive
Andrewes et al [45]	Time based	6	No	6	15	8	15	Smartphone	Fixed
Berenson et al [24]	Time based	21	No	5	8	N/R^h^	10	Others	N/R
Briones-Buixassa et al [46]	Time and event based	15	No	3	N/R	N/R	15	Smartphone	Fixed
Chaudhury et al [47]	Time based	7	No	6	N/R	N/R	N/R	Others	N/R
Coifman et al [48]	Time based	21	No	5	N/R	7.5	10	Others	Incremental
Ebner-Priemer et al [49]	Time based	14	No	N/R	N/R	N/R	N/R	Others	N/R
Ellison et al [50]	Time and event based	21	Yes	6	6	5	N/R	Smartphone	Incremental
Hawkins et al [51]	Time based	14	Yes	5	N/R	N/R	N/R	Others	Fixed
Houben et al [52]	Time based	8	No	10	N/R	N/R	N/R	Others	No incentive
Kaurin et al [53]	Event based	21	No	N/R	26	N/R	N/R	Others	Incremental
Köhling et al [54]	Time based	7	No	5	18	N/R	N/R	Smartphone	Fixed
Lane et al [55]	Time and event based	21	Yes	6	N/R	N/R	N/R	Others	Incremental
Links et al [16]	Time based	21	No	6	N/R	N/R	N/R	Others	Fixed
Moukhtarian et al [56]	Time based	5	No	8	7	2	16	Others	Fixed
Rizk et al [57]	Time based	7	No	6	9	N/R	N/R	Others	N/R
Santangelo et al [12]	Time based	4	No	12	6	N/R	N/R	Others	Incremental
Scala et al [58]	Time based	21	No	6	N/R	N/R	N/R	Others	N/R
Selby et al [59]	Time and event based	14	No	5	32	N/R	N/R	Smartphone	Incremental and fixed
Solhan et al [60]	Time based	28	No	6	21	N/R	15	Others	Incremental
Southward et al [61]	Time based	2	No	12	N/R	N/R	N/R	Smartphone	N/R
Stiglmayr et al [14]	Time based	2	No	12	32	N/R	N/R	Others	N/R
Trull et al [17]	Time based	28	No	6	37	N/R	10	Others	Incremental
Veilleux et al [62]	Time and event based	7	Yes	7	N/R	2	10	Smartphone	Fixed
Weise et al [63]	Time based	4	N/R	N/R	N/R	N/R	N/R	Smartphone	N/R
Wright et al [70]	Event based	21	N/R	N/R	87	2	N/R	Smartphone	N/R
Wycoff et al [64]	Time and event based	N/R	Yes	N/R	N/R	N/R	N/R	Others	Incremental
Zaehringer et al [65]	Time based	4	No	12	17	N/R	N/R	Smartphone	Fixed
Zaki et al [66]	Time based	21	No	5	N/R	N/R	N/R	Others	Incremental

^a^Time based refers to the format in which the prompts are sent to the participants according to a fixed or random scheme within a window of time, for example, every 2 hours during walking time. Event based refers to when participants are asked to self-initiate a prompt in X situation, for example, when they have a suicidal thought.

^b^Total number of days of the study.

^c^A daily extra measurement in addition to the prompts.

^d^Number of prompts sent each day of the study.

^e^Items to be answered in each prompt.

^f^Estimate of minutes required to complete each prompt.

^g^Number of minutes for which the prompt was available before it was considered lost.

^h^N/R: not reported.

### Quality of the Included Studies

The total quality scores of the eligible studies ranged from 5 (4/28, 14%) to 8 (8/8, 29%) out of 9 (total scores; refer to Tables 3 and 4). A total of 36% (10/28) of studies were of moderate quality, 29% (8/28) of studies were of high quality, and none of the included studies were rated as having low quality. All studies met the quality criterion “Ascertainment of Exposure” of the selection dimension and the criteria “Assessment of Outcome,” “Follow-up Long Enough for Outcomes to Occur,” and “Adequacy of Follow-up of Cohorts” of the outcome dimension, but the quality criterion “Demonstration of Absence of Outcome at Baseline” was met by none of the included studies.

**Table 3:** Quality assessment of the included studies—representativeness of the exposed cohort, Selection of nonexposed group, ascertainment of exposure, and demonstration of absence of outcome at baseline.

Study	Selection			
	Representativeness of the exposed cohort	Selection of nonexposed group	Ascertainment of exposure	Demonstration of absence of outcome at baseline
Andrewes et al [45]	1	N/A^a^	1	0
Berenson et al [24]	1	1	0	1
Briones-Buixassa et al [46]	1	1	0	0
Chaudhury et al [47]	1	N/A	1	0
Coifman et al [48]	1	1	1	0
Ebner-Priemer et al [49]	1	1	0	0
Ellison et al [50]	1	1	1	0
Hawkins et al [51]	1	1	0	0
Houben et al [52]	1	1	1	0
Kaurin et al [53]	1	1	1	0
Köhling et al [54]	1	1	1	0
Lane et al [55]	1	1	0	0
Links et al [16]	1	N/A	1	0
Moukhtarian et al [56]	1	0	1	0
Rizk et al [57]	1	N/A	1	0
Santangelo et al [12]	0	0	1	0
Scala et al [58]	1	1	1	0
Selby et al [59]	1	1	1	0
Solhan et al [60]	1	1	1	0
Southward et al [61]	1	N/A	1	0
Stiglmayr et al [14]	1	1	1	0
Trull et al [17]	1	1	1	0
Veilleux et al [62]	1	1	1	0
Weise et al [63]	1	N/A	1	0
Wright et al [70]	1	N/A	1	0
Wycoff et al [64]	1	N/A	1	0
Zaehringer et al [65]	0	N/A	1	0
Zaki et al [66]	1	1	1	0

^a^N/A: not applicable to the respective study owing to the lack of a control group.

**Table 4:** Quality assessment of the included studies—comparability based on the design or analysis, assessment of outcome, follow-up enough long for outcomes to occur, and adequacy of follow-up of cohorts.

Study	Comparability based on the design or analysis	Outcome			Total quality score
		Assessment of outcome	Follow-up long enough for outcomes to occur	Adequacy of follow-up of cohorts	
Andrewes et al [45]	0	1	1	1	5
Berenson et al [24]	1	1	1	1	7
Briones-Buixassa et al [46]	0	1	1	1	5
Chaudhury et al [47]	1	1	1	1	6
Coifman et al [48]	2	1	1	1	8
Ebner-Priemer et al [49]	0	1	1	1	5
Ellison et al [50]	2	1	1	1	8
Hawkins et al [51]	0	1	1	1	5
Houben et al [52]	2	1	1	1	8
Kaurin et al [53]	0	1	1	1	
Köhling et al [54]	2	1	1	1	8
Lane et al [55]	0	1	1	1	5
Links et al [16]	1	1	1	1	6
Moukhtarian et al [56]	1	1	1	1	6
Rizk et al [57]	1	1	1	1	6
Santangelo et al [12]	1	1	1	1	5
Scala et al [58]	2	1	1	1	8
Selby et al [59]	0	1		1	6
Solhan et al [60]	2	1	1	1	8
Southward et al [61]	0	1	1	1	5
Stiglmayr et al [14]	1	1	1	1	7
Trull et al [17]	2	1	1	1	8
Veilleux et al [62]	0	1	1	1	6
Weise et al [63]	1	1	1	1	6
Wright et al [70]	1	1	1	1	6
Wycoff et al [64]	0	1	1	1	5
Zaehringer et al [65]	2	1	1	1	6
Zaki et al [66]	2	1	1	1	8

### Narrative Review

#### Overview

A total of 28 articles that met the eligibility criteria were included in the narrative review. All selected studies used EMA to investigate certain aspects of BPD psychopathology. Table 5 shows the descriptive statistics of the study variables for all the studies.

**Table 5:** Descriptive statistics of the included studies (n=28).

Characteristics		Values
Age (years; n=27), mean (SD)		28.58 (3.74)
Study length (n=28), mean (SD)		13.32 (8.61)
Prompts per day (n=24), mean (SD)		6.917 (2.64)
Number of items (n=14), mean (SD)		15.88 (10.37)
Required answer time (minutes; n=6), mean (SD)		4.41 (2.83)
Assessment window (minutes; n=10), mean (SD)		22.10 (20.12)
Dropout of participants with BPD^a^ (n=8), mean (SD)		8.91 (10.15)
Quality score (n=28), mean (SD)		6.36 (1.19)
Sex (female; n=27 studies), %		86.1
**Location (n=28), n (%)**		
	Europe	8 (29)
	Oceania	1 (4)
	South America	1 (4)
	United States	17 (61)
	United States and Europe	1 (4)
**Sampling scheme (n=28), n (%)**		
	Time based	20 (71)
	Event based	2 (7)
	Both	6 (21)
**Instrument (n=28), n (%)**		
	DSM^b^	26 (93)
	ICD^c^	2 (7)
**Inpatient (n=28), n (%)**		
	Yes	3 (11)
	No	25 (89)
**Extra assessment (n=26), n (%)**		
	Yes	5 (19)
	No	21 (81)
**Device type (n=28), n (%)**		
	Smartphone	11 (39)
	Others	17 (61)
**Fixed increment (n=19), n (%)**		
	Fixed	8 (42)
	Incremental	9 (47)
	Both	1 (5)
	No incentive	1 (5)
**Quality rating (n=28), n (%)**		
	Low	0 (0)
	Moderate	18 (64)
	High	10 (36)

^a^BPD: borderline personality disorder.

^b^DSM: Diagnostic and Statistical Manual for Mental Disorders.

^c^ICD: International Classification of Diseases.

#### Participant Characteristics

The average number of participants across the studies included in the systematic review was 42.88 (range 5-107). Of the 28 included studies, 27 (96%) reported the sex of the participants. The average proportion of female participants was 86.1%, whereas 25% (7/28) of studies recruited only female participants. Excluding these studies, the average proportion of female participants was 62.4% across the studies. The mean age of participants was 28.58 (SD 3.74) years. Overall, 11% (3/28) of studies were conducted among inpatients, and 89% (25/28) of studies recruited participants from community or nonclinical settings. In total, 54% (15/28) of studies investigated mixed BPD features, whereas 32% (9/28) of studies examined only affective dysregulation, 11% (3/28) of studies examined only behavioral dysregulation features, and only 4% (1/28) of studies examined cognitive or self-disturbance features.

#### Study Characteristics

##### Study Length (Measurement Period)

The length of the EMA studies ranged from 2 to 28 days, with an average of 13.32 (SD 8.61) days. A total of 43% (12/28) of studies conducted EMA for <10 days, including studies that delivered prompts for 2 days (2/28, 7%), 4 days (3/28, 11%), 5 days (1/28, 4%), 6 days (1/28, 4%), 7 days (4/28, 14%), and 8 days (1/28, 4%). A total of 54% (15/28) of studies conducted EMA for >10 days, with 11% (3/28) of studies conducting EMA for 14 days, 4% (1/28) of studies for 15 days, and 32% (9/28) of studies for >21 days. A total of 7% (2/28) of studies conducted EMA for 28 days, and only 4% (1/28) of studies did not report these data. In 18% (5/28) of studies, an extra EMA assessment was delivered each day to the participants in addition to prompts.

##### Sampling Strategy

A total of 71% (20/28) of studies used only time-based sampling protocols, 21% (6/28) of studies used a combination of both time-based and event-based sampling protocols, and only 7% (2/28) of studies used only event-based sampling protocols.

##### Sampling Frequency (Number of Daily Prompts)

Of the 28 studies included in the systematic review, 24 (86%) studies reported the prompting frequency. The average prompting frequency was 6.92 (SD 2.6; range 3-12) times per day. Most studies (14/28, 50%) reported prompting participants <10 times a day, with 4% (1/28) of studies reporting prompting participants 10 times a day and 14% (4/28) of studies reporting prompting participants 12 times a day.

##### Number of Items

The average number of items per prompt was 15.88 (SD 10.37; range 6-87) across studies. A total of 50% (14/28) of studies reported the actual number of items, with 18% (5/28) of studies assessing 6 to 9 items, 11% (3/28) of studies assessing 15 to 18 items, 7% (2/28) of studies assessing 21 to 26 items, 11% (3/28) of studies assessing 32 to 37 items, and 4% (1/28) of studies assessing 87 items.

##### Required Answer Time

Only 21% (6/28) of studies reported the required answer time. Across these studies, the average time to answer a prompt was 4.41 (SD 2.83; range 2-8) minutes.

##### Response Window

A total of 36% (10/28) of studies indicated the response or assessment window, with an average assessment window of 22.10 (SD 20.12; range 10-16) minutes per prompt.

##### Sampling Devices (Equipment Used)

All the included studies (28/28, 100%) reported their sampling device. A total of 39% (11/28) of studies used a smartphone as the EMA device type, whereas other studies (17/28, 61%) used different device tools, such as PDA (5/28, 18%), Tungsten E Palmplot (Palm Inc; 3/28, 11%), handheld electronic organizer (3/28, 11%), Apple iPods (Apple Inc; 1/28, 4%), Palmplot computer (Palm Inc; 1/28, 4%), Motorola Razr (Motorola Inc) with Android (Google LLC; 1/28, 4%), Palm Zire 31 (Palm Inc; 2/28, 7%), and handheld Zire 21 (Palm Inc; 1/28, 4%).

##### Financial Incentives for Participants

A total of 68% (19/28) of studies reported their incentive scheme. In 32% (9/28) of studies, the financial incentive was incremental; in 29% (8/28) of studies, it was fixed; 4% (1/28) of studies reported a mixed incentive scheme (fixed and incremental); and only 4% (1/28) of studies reported not using a financial incentive.

##### Dropout Rate for the BPD Participants

Only 25% (7/28) of studies reported the dropout rate of the participants with BPD. Of these, 7% (2/28) of studies reported no dropouts. The remaining 18% (5/28) of studies reported a dropout rate of 1.9% to 30%. The average dropout rate was 8.91% (SD 10.15%).

### Meta-analysis

#### Overview

For the meta-analytical synthesis of compliance rates and predictors, we could only use a subset of 18 (64%) studies [12,16,17,45-48,50,52,54,56,58,60-62,64-66] drawn from the initial sample of 28 studies. This subset included all studies that used EMA to examine BPD psychopathology and also provided sufficient information that allowed the estimation of an average compliance rate for BPD or BPD features. Thus, 36% (10/28) of studies that did not provide information about compliance had to be excluded from the meta-analysis.

#### Compliance Rate

**Figure 2:**
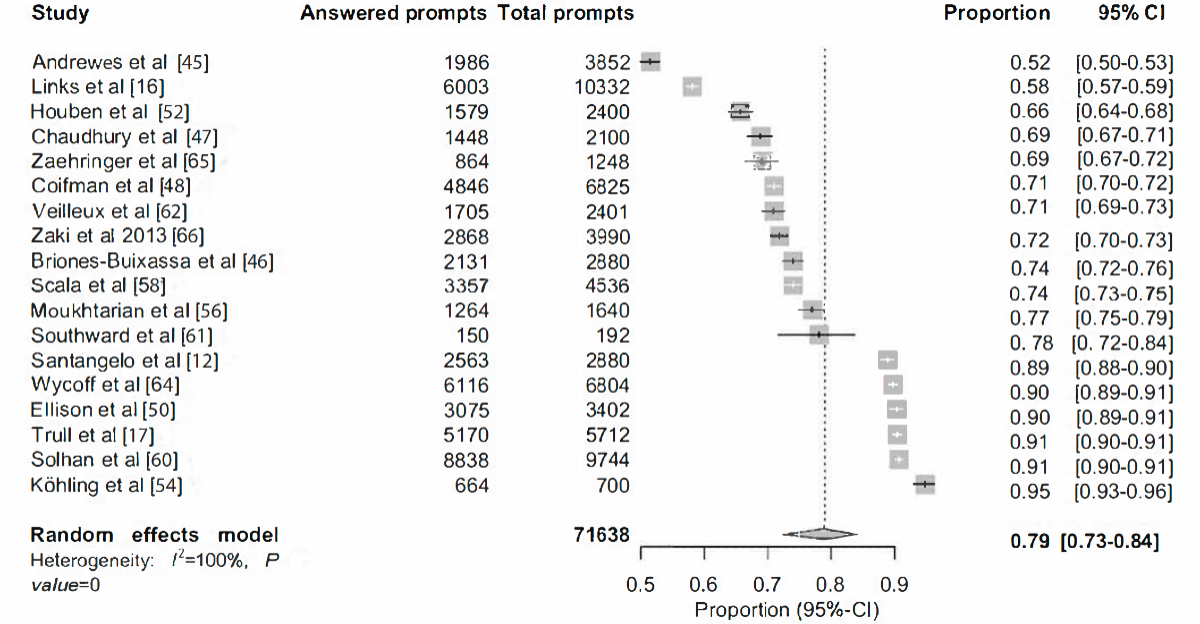
The pooled rate for compliance among participants with borderline personality disorder.

The pooled prevalence rate of compliance among BPD participants was 79% (95% CI 0.73-0.84; Figure 2). Almost complete between-study heterogeneity was observed (*I*^2^=99.7%*,* *P*<.001).

#### Potential Predictors of Compliance

In our meta-analysis, we were able to model the predictive value of 5 specific design features and procedures of EMA studies in the subgroup analysis for categorical variables (ie, diagnostic criteria for selection of participants with BPD, sampling scheme, device type, extra assessment, and total quality score) on pooled rates of compliance. None of the predictors were found to be significant in the subgroup analysis with weighted effect sizes (Table 6).

**Table 6:** Subgroup analyses of compliance rates for categorical moderator variables.

			Compliance rate (95% CI)							Heterogeneity test				
			Studies, n (%)		Compliance rate (%)		95% CI		*Q* value			*I* ^2^		*P* value
**Diagnostic criteria**									0.39					.53
	DSM^a^	16 (57)		78.36		71.37-84.02					99.7			
	ICD^b^	2 (7)		83.8		64.34-93.68					99.4			
**Sampling scheme**									0.64					.42
	Time based	14 (50)		77.73		70.13-83.84					99.8			
	Time and event based	4 (14)		83.09		70.12-91.15					99.5			
**Device type**									0.00					.96
	Smartphone	7 (25)		78.81		70.70-85.67					99.6			
	Other	11 (39)		79.16		67.72-86.84					99.7			
**Quality rating**									2.89					.09
	Low	N/A^c^		N/A		N/A					N/A			
	Moderate	10 (36)		74.45		65.16-81.95					99.6			
	High	8 (29)		83.84		75.96-89.49					99.7			
	**Extra assessment**							1.32					.25	
	Yes	3 (11)		85.5		71.83-93.17					99.4			
	No	15 (54)		77.49		70.27-83.37					99.7			

^a^DSM: Diagnostic and Statistical Manual for Mental Disorders.

^b^ICD: International Classification of Diseases.

^c^N/A: not applicable.

#### Predictors With Missing Data

For 4 relevant variables (ie, number of items, required answer time, response window, and dropout rate), 43% (12/28) of studies did not report sufficient information to extract relevant data (Tables 1 and 2). Only 29% (8/28) of studies reported the actual number of items, which ranged from 6 to 37. Only 18% (5/28) of studies reported the required answer time, which ranged between 2 and 8 minutes, and only 25% (7/28) of studies indicated the response window, which ranged from 10 to 16 minutes. Finally, only 25% (7/28) of studies reported the dropout rate, which ranged between 0% and 30% across studies.

## Discussion

### Principal Findings

To our knowledge, this is the first systematic review, narrative review, and meta-analysis describing EMA study design features and procedures as they are used in current BPD research and estimating the effects of EMA study features and procedures on the compliance rates of participants diagnosed with BPD. The aim of the narrative review was to describe the characteristics of EMA protocols (study features and procedures) with patients with BPD using a systematic review approach. High heterogeneity was found with respect to study features and procedures across the different studies. Our results regarding the heterogeneity of features and procedures in BPD research using EMA are in line with the general trends in broad EMA research identified previously by Wrzus and Neubauer [26] and Vachon et al [27]. Considering the above, we can point out that similar pitfalls and challenges related to such heterogeneity apply to EMA studies in BPD. These challenges are primarily related to the problem of integrating and aggregating data stemming from studies that use very different design features and procedures. In short, similar to the broad EMA use in health research, a more homogeneous set of features and procedures could allow for more direct comparisons and benefit the overall advancement of the field, further highlighting the benefits of using EMA for BPD psychopathology research.

Considering the need for more standardized EMA designs and the crucial importance of compliance rates to obtain robust data sets in EMA research of BPD, the aim of the meta-analysis was to estimate the effects of specific design features and procedures on participant compliance rates. Across the studies included in the meta-analysis, the pooled prevalence rate for compliance among participants with BPD was estimated to be 79%, which is very similar to the 80% compliance rate recommended by Stone and Shiffman [8]. This suggests that, on average, EMA research is feasible for participants with BPD. Nevertheless, the reported compliance rates were high across the included studies and varied from 52% [45] to 95% [54]. This high variability suggests that some design features or procedures may have a bearing on compliance rates and thus justifies a specific exploration of such potential effects. However, contrary to our predictions, we could not identify the features or procedures of EMA designs that have an effect on compliance. It is important to note that only a small set of studies was included in the analysis, and only a limited number of design features and procedures could be statistically tested. On the basis of an analysis of the available design features and procedures, study characteristics, and outcomes, it is possible that compliance is robust and stable across varying EMA prompt calibrations and designs with participants with BPD. Moreover, our analysis suggests that compliance of participants with BPD appears to exhibit significant tolerance for a variety of design features and procedures of EMA studies, including prompt intensities, the measurement period of the study, the number of items in each prompt, the estimated time for the completion of each prompt, and the time window from the prompt signal to answering the prompt. No effect was found for other relevant variables such as the nature of the incentive for participants and technology used (device type). However, further investigation is warranted once more studies are available. In such studies, it would be useful if authors would add additional information regarding procedures and design features, including the methods for enhancing participation and compliance, such as phone calls, intensive monitoring, and variable incentives. It would also be essential for future EMA research of BPD to have more standardized criteria for the reporting of the technical aspects, design features, and procedures. Such standardized criteria would certainly allow for a more focused and comprehensive testing of variables that may have a bearing on compliance. Thus, EMA design can be strengthened and yield a full range of benefits to our field of study.

### Future Directions for EMA Studies in BPD: Can We Agree on the Reporting of Basic Design Features and Procedures of EMA Studies?

The third aim of this study was to provide recommendations to foster compliance by choosing design features and procedures of EMA that can enhance compliance for future studies that adopt EMA for research in BPD psychopathology [67]. Our study found that EMA studies use vastly varying setups. These include divergent within-day prompt frequencies, measurement periods, compensation schedules, number of items, required answering times, assessment windows, device types, and extra assessment plans. EMA is extremely useful for understanding complex dynamics in BPD psychopathology; however, the variety of designs and setups currently preclude the aggregation of results across studies and learning which study setups are best to foster compliance. Thus, it is rather difficult to draw stable conclusions regarding EMA findings. Shiffman and Stone [9] have established some general guidelines for designing and reporting in EMA studies, but they did not provide recommendations for the actual reporting of the features and procedures of EMA designs in the studies or in supplemental materials. Some reporting guidelines have been widely adopted in other types of studies. For example, STROBE (Strengthening the Reporting of Observational Studies in Epidemiology) [68] is a well-known checklist for observational studies. It consists of 22 items related to each section of the article (ie, title, abstract, introduction, methods, results, and discussion) with the aim of improving the quality of the reports. Following the same principle, a recent systematic review of methods and procedures used in EMA of nutrition and physical activity research in youths [69] developed a comprehensive checklist of specific items to be reported in EMA studies: Checklist for Reporting EMA Studies. More recently, Trull et al [71] made an effort to fill the gap of standardization in how data and procedures are reported in EMA studies related to psychopathology and published a review of recommended reporting guidelines and current practices for ambulatory assessment research in psychopathology. The authors reviewed publications from the last 7 years (2012-2018) from 3 major mental health journals (*Journal of Abnormal Psychology, Psychological Medicine,* and *Clinical Psychological Science*) and integrated the information on how the various studies reported the methods used in EMA with some previously existing guidelines from EMA study reports, making an effort to provide some recommendations for future EMA studies in mental health. Despite the growing number of EMA studies in BPD, there is still a lack of such specific guidelines. Santangelo et al [67] have already made an effort to synthesize the EMA literature on BPD symptomatology. They discussed the different characteristics of EMA studies, providing an overview of EMA findings in BPD based on DSM-IV criteria. The authors also mentioned different challenges of EMA and provided recommendations to enhance them. However, they did not specifically focus on the technical aspects of EMA as this study did. It is likely that to produce specific recommendations, as suggested by Santangelo et al [67], different setups need to be compared with each other in terms of their relative benefits for compliance, including additional variables and procedures used to enhance compliance. Therefore, standard reporting of design features and procedures of EMA studies is required to fully understand how to optimize participation and obtain robust data sets that can then be combined and compared across EMA studies that examine BPD psychopathology.

### Limitations

Although this study is innovative and unique in its contribution to EMA research on BPD psychopathology, the results must be interpreted with certain limitations in mind. First, despite the fact that there are numerous published studies that use EMA to investigate BPD (we found 79 studies in our literature search), only approximately one-third of all published studies (28 of the 79 studies identified in our literature search, 35%) that use EMA for BPD psychopathology actually provide specific information that allows us to extract relevant design features and procedures. Thus, our narrative review was based on a limited number of studies and a limited number of design features and procedures.

Second, available studies were even fewer when we attempted to quantitatively analyze the predictors of compliance rates. Only about a quarter of all studies identified (18 of the 79 studies identified in the search, 23%) provided enough information to calculate compliance rates (eg, they provided the theoretical number of prompts to be answered and the actual average number of answered prompts among participants in the study). Trull et al [71] have already warned that systematic evaluation and aggregation of the findings of EMA studies by meta-analysis critically depends on the clear and explicit reporting of study characteristics. Among the studies included in this review, most studies did not report several design features and procedures of EMA, including the number of items, required answer time, assessment window, and dropout. Therefore, we were unable to perform quantitative analyses for these variables using the included studies to examine their influence on compliance.

Third, we found high heterogeneity in compliance rates, which was not explained by selected predictors (ie, study design features and procedures) or subgroup analyses (ie, age groups and sex). Furthermore, we were not able to examine the effect of patient-level data in our meta-regression analysis (ie, percentage of females and mean age of sample) because the results of a meta-regression based on the study means of patient-level variables are prone to bias (ie, ecological fallacy) [72].

Fourth, additional variables that were not examined in our study may have an influence on compliance. For example, it is possible that monitoring compliance and calling participants to improve participation may foster compliance. An important point to be noted is that there are only a few studies that we could include because other studies lacked the design variables examined in this systematic review and meta-analysis. In addition, within the included studies, a considerable amount of key information to be analyzed as moderators of compliance was missing (eg, the number of items and required answer time).

Finally, regarding compliance, there may be a systematic publication bias. Studies with very low compliance likely do not yield valid results, so publication is reasonably withheld. This means that there may be a restriction in the reported range of compliance rates. Therefore, we can say that the conclusions of this study apply to EMA setups that have been relatively successful in securing participation across studies, which, by definition, raises the average compliance rates. Indeed, much could be learned from studies that did not yield acceptable compliance rates, but this literature is typically not available in published form.

### Conclusions

This systematic review and meta-analysis described the design features and procedures used in EMA studies investigating BPD and reported that overall compliance rates among the included studies appeared to be stable and acceptable across different EMA calibrations and study designs. In addition, our findings emphasize the importance of moving toward standardized reporting of EMA designs (eg, prompt frequency) and findings (eg, variability indices) to improve and aggregate our knowledge of basic BPD psychopathology research using EMA. As early indications are that compliance might be robust and stable across varying setups, we (1) propose that future EMA studies should consider other relevant design features and procedures, such as indices of variability, when designing EMA protocols to investigate BPD; (2) hypothesize that there may be nonspecific factors of EMA study design that may have an impact on compliance rates, which should be addressed in future EMA studies of patients with BPD; and finally, (3) suggest that standard reporting of the design features and procedures of EMA is thus required for future studies.

## Appendix

Multimedia Appendix 1 PRISMA (Preferred Reporting Items for Systematic Reviews and Meta-Analyses) checklist.

Multimedia Appendix 2 Search terms used in the individual databases.

## Abbreviations

BPD: borderline personality disorder
DSM: Diagnostic and Statistical Manual for Mental Disorders
EMA: ecological momentary assessment
MOOSE: Meta-Analyses of Observational Studies in Epidemiology
PRISMA: Preferred Reporting Items for Systematic Reviews and Meta-Analyses
STROBE: Strengthening the Reporting of Observational Studies in Epidemiology

## Data Availability

Data will be made available upon reasonable request from the authors.
