# Supplementary material for: Study Features and Response Compliance in Ecological Momentary Assessment Research in Borderline Personality Disorder: Systematic Review and Meta-analysis
Source: J Med Internet Res. 2023 Mar 15;25:e44853. doi: 10.2196/44853 (PMC10131785; doi:10.2196/44853)
Supplement: Multimedia Appendix 2 [file jmir_v25i1e44853_app2.docx]

| **eTable 1**  *Search strategy* | | |
| --- | --- | --- |
| **OR**  **OR**  **OR**  **OR**  **OR** | **1** | **2** |
|  | **AND** | |
|  | “ecologic* momentary assessment” OR “event contingent recording” OR “experience sampling” or “ecological momentary intervention” OR “intensive time sampling” OR “daily diary methods” OR “electronic diar*” OR “ambulatory monitoring” OR “ambulatory assessment” OR “intensive diar*” | “borderline personality disorder” OR “borderline personality” OR “borderline personality characteristics” OR “borderline personality features” OR “borderline personality symptoms” OR “emotionally unstable personality disorder” |
|  |  | “emerging personality disorder” OR “personality disorder” |
|  |  | “non suicidal self injur*” OR parasuicide OR “parasuicidal behavio#r” OR “parasuicidal gestures” OR “parasuicidal behavio#r ideation” OR “suicidal ideation” |
|  |  | “affect* dysregulation” OR “affect* instability” OR “instability of self-esteem” OR “self-esteem dysregulation” OR “dissociative symptom*” OR “dissociative pattern*” OR “self-concept clarity” |
|  |  | “interpersonal conflict*” OR “interpersonal conflict*” OR “interpersonal problem*” OR “rejection sensitivity” |

## **Multimedia Appendix 2:** Search terms used in the individual databases.
